# α-Ionone, an Apocarotenoid, Induces Plant Resistance to Western Flower Thrips, *Frankliniella occidentalis*, Independently of Jasmonic Acid

**DOI:** 10.3390/molecules25010017

**Published:** 2019-12-19

**Authors:** Mika Murata, Tetsuya Kobayashi, Shigemi Seo

**Affiliations:** 1 Institute of Vegetable and Floriculture Sciences, National Agriculture and Food Research Organization, Tsu, Mie 514-2392, Japan; muratam@affrc.go.jp; 2 Institute of Agrobiological Sciences, National Agriculture and Food Research Organization, Tsukuba, Ibaraki 305-8602, Japan; ttkoba@affrc.go.jp

**Keywords:** Thripidae, western flower thrips, Noctuidae, common cutworm, apocarotenoid, α-ionone, herbivore resistance, tomato, *Arabidopsis*, jasmonic acid

## Abstract

Apocarotenoids, such as β-cyclocitral, α-ionone, β-ionone, and loliolide, are derived from carotenes via chemical or enzymatic processes. Recent studies revealed that β-cyclocitral and loliolide play an important role in various aspects of plant physiology, such as stress responses, plant growth, and herbivore resistance. However, information on the physiological role of α-ionone is limited. We herein investigated the effects of α-ionone on plant protection against herbivore attacks. The pretreatment of whole tomato (*Solanum lycopersicum*) plants with α-ionone vapor decreased the survival rate of western flower thrips (*Frankliniella occidentalis*) without exhibiting insecticidal activity. Exogenous α-ionone enhanced the expression of defense-related genes, such as basic β-1,3-glucanase and basic chitinase genes, in tomato leaves, but not that of jasmonic acid (JA)- or loliolide-responsive genes. The pretreatment with α-ionone markedly decreased egg deposition by western flower thrips in the JA-insensitive *Arabidopsis* (*Arabidopsis thaliana*) mutant *coi1-1*. We also found that common cutworm (*Spodoptera litur*a) larvae fed on α-ionone-treated tomato plants exhibited a reduction in weight. These results suggest that α-ionone induces plant resistance to western flower thrips through a different mode of action from that of JA and loliolide.

## 1. Introduction

Attacks by herbivore pests cause severe losses in crop yield and quality. Insecticides have been widely used for pest control because of their highly effective and rapid action on targets. However, the repeated use of a synthetic insecticide often causes the appearance of resistant herbivore pests [1]. Therefore, there are constant needs for the development of new agrochemicals that overcome pest resistance to insecticides. Some of these agrochemicals are compounds that activate the defense responses of plants to herbivore pests and do not exhibit direct insecticidal activity [2,3]. A well-known example is the phytohormone jasmonic acid (JA), which plays an important role in herbivore resistance [4]. However, information on these compounds, other than JA and JA-related compounds, is limited.

We recently demonstrated that loliolide induced resistance to multiple herbivore pests, such as the two-spotted spider mite (*Tetranychus urticae*), western flower thrips (*Frankliniella occidentalis*), and common cutworm (*Spodoptera litura*), through the activation of JA-independent defense responses [5]. Loliolide has also been shown to function as an allelochemical in plant–plant communications [6,7]. Loliolide is produced via the chemical or enzymatic degradation process of α-carotene and β-carotene [5,8,9,10]. The degradation of α- and β-carotenes also results in the production of other apocarotenoids, such as β-cyclocitral, α-ionone, and β -ionone [11]. β-cyclocitral has been shown to play an important role in various aspects of plant physiology. Molecular genetic analyses demonstrated that β-cyclocitral regulated the plant response to environmental stresses, such as oxidative stress and strong light, and root growth in *Arabidopsis* [12,13,14,15]. Furthermore, the exogenous application of β-cyclocitral inhibited infestation by the two-spotted spider mite (*T. urticae* Koch) in African spider plants and reduced disease symptoms caused by the plant pathogenic oomycete *Plasmopara viticola* in grapevines [16,17]. β-ionone is a major scent compound emitted from plants and has been widely used as a fragrant material in the cosmetic and food industries [18]. Analyses of *Arabidopsis* (*Arabidopsis thaliana*) overexpressing carotenoid cleavage dioxygenase1 (*CCD1*), a gene involved in the synthesis of apocarotenoids, revealed that β-ionone exerted repellent effects on the crucifer flea beetle (*Phyllotreta cruciferae* Goeze), two-spotted spider mite, and silverleaf whiteflies (*Bemisia tabaci* Gennadius) [19,20]. Although α-ionone is also a scent compound, its physiological activities for plants and its physiological roles in plants remains unclear. 

In the course of investigating apocarotenoids capable of protecting plants against herbivores, we found that α-ionone was effective for western flower thrips. We herein demonstrated that the exogenous application of α-ionone-induced plant resistance to western flower thrips without exhibiting insecticidal activity.

## 2. Results

### 2.1. α-Ionone Decreases the Survival Rate of Western Flower Thrips Without Exhibiting Insecticidal Activity

**Figure 1:**
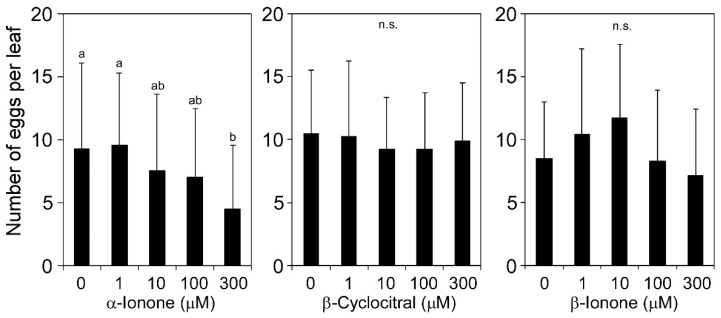
Effects of apocarotenoids on the infestation of tomato leaves by western flower thrips. Tomato leaves treated with different concentrations of α-ionone, β-cyclocitral, or β-ionone for 48 h were used for the infestation assay using western flower thrips. Female thrips were placed on leaf disks, and the numbers of laid eggs were counted 3 d after the inoculation. Values are the mean ± SD (*n* = 22–26 replicates). Different letters indicate significant differences among treatments (Tukey–Kramer honestly significant difference (HSD) test, *p* < 0.05).

To examine the effects of apocarotenoids on herbivores, we released adult female western flower thrips onto micro-tom tomato (*Solanum lycopersicum*) leaves that were treated with β-cyclocitral, α-ionone, or β-ionone. Since loliolide effectively decreased the susceptibility to western flower thrips at 300 μM when applied to *Arabidopsis* leaves [5], we used concentrations of 300 μM or lower for β-cyclocitral, α-ionone, and β-ionone. The treatment with α-ionone at 300 μM decreased the number of eggs laid by the pest (Figure 1; *p* < 0.05, *F* = 2.800). Neither β-cyclocitral nor β-ionone exerted this inhibitory effect on egg deposition (*p* > 0.05, *F* = 0.162 for β-cyclocitral; *p* > 0.05, *F* = 1.163 for β-ionone). We focused on α-ionone in subsequent experiments. 

**Figure 2:**
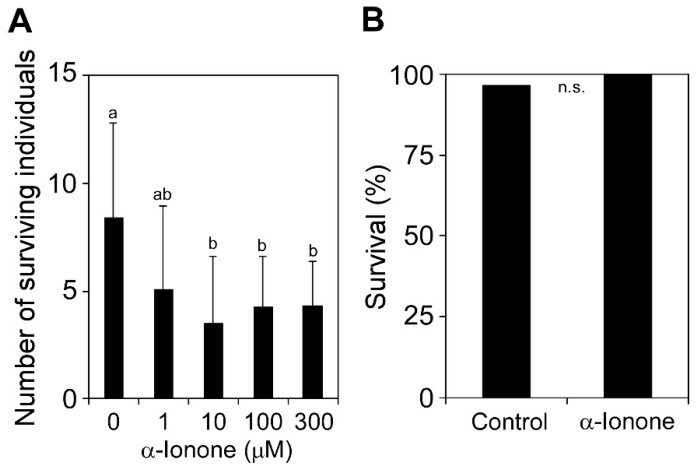
Effects of α-ionone on the infestation of tomato leaves by western flower thrips and insecticidal activities for thrips. (**A**) Tomato plants were exposed to different concentrations of α-ionone for 24 h, and female thrips were released on the treated plant. The numbers of surviving individuals were counted 14 d after the inoculation. Values are the mean ± SD (*n* = 11–15 replicates) Different letters indicate significant differences among treatments (Tukey–Kramer HSD test, *p* < 0.005). (**B**) Insecticidal activity assay. The numbers of surviving larvae 48 h after dipping into a solution containing 300 μM α-ionone or solvent alone (control) were counted (*n* = 30 larvae, *t*-test, *p* > 0.05).

To examine whether exogenous α-ionone inhibits the survival of western flower thrips, we released female thrips onto the leaf surface of tomato plants that were treated with α-ionone vapor for 24 h (Supplemental Figure S1). Since α-ionone vapor treatment of tomato plants for 48 h or longer in a sealed pot caused water droplets on their leaf surface, we assayed with a 24 h treatment. α-Ionone at 10 μM or higher decreased the survival rate of female thrips (Figure 2A; *p* < 0.005, *F* = 4.560). To examine whether this decrease in the survival rate was due to the direct insecticidal activity of α-ionone for western flower thrips, we performed an assay to assess the toxicity of insecticides. When female mites were dipped into a solution containing 300 μM α-ionone or 0.1% methanol as a control, no significant differences were observed in survival rates between treatments (Figure 2B; *p* = 1.000).

### 2.2. α-Ionone-Induced Thrips Resistance Occurs Independently of JA

**Figure 3:**
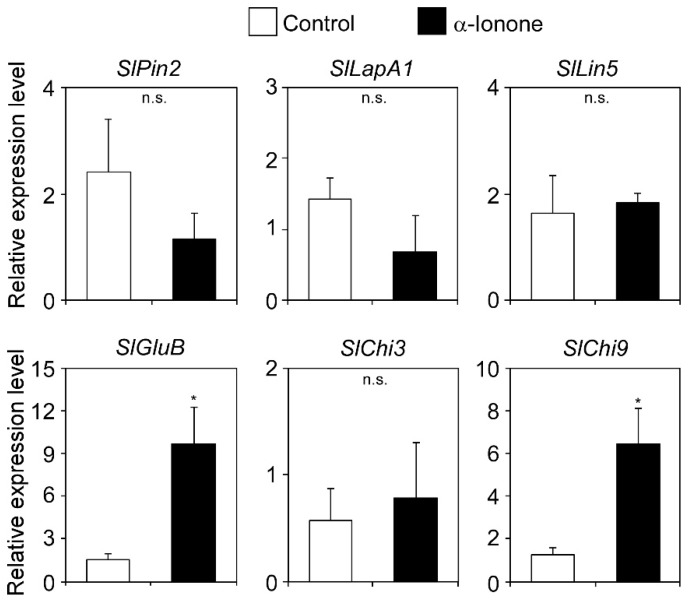
Gene expression analysis of tomato plants after a treatment with α-ionone vapor. Real-time PCR analysis of the indicated genes in tomato leaves 24 h after exposure to 300 μM α-ionone or solvent alone (control). Values are the mean ± SD (*n* = three replicates; * *p* < 0.05, *t*-test).

The above results suggested that reductions in the survival of western flower thrips and egg deposition by the same pest on tomato leaves were due to host defense responses induced by exogenously applied α-ionone. To gain insights into the α-ionone-induced defense mechanism, we analyzed the induction kinetics of herbivore-responsive tomato genes, such as those encoding proteinase inhibitor II (*SlPin2*) [21], leucine aminopeptidase (*SlLapA1*) [22], acidic chitinase (*SlChi3*) [23], basic chitinase (*SlChi9*) [23], basic β-1,3-glucanase (*SlGluB*), and cell-wall invertase (*SlLin5*) [5]. Tomato plants were exposed to α-ionone vapor for 24 h, and the expression levels of these marker genes in the treated leaves were examined by real-time PCR. α-Ionone enhanced the expression of *SlChi9* and *SlGluB* (Figure 3). The *SlPin2*, *SlLapA1*, *SlChi3*, and *SlLin5* genes were not induced by α-ionone. A quantitative analysis of endogenous JA in tomato leaves showed that JA levels were not changed by the treatment with α-ionone vapor (Supplementary Figure S2). Since the expression of *SlGluB* is known to be induced by salicylic acid (SA) [24,25], we also measured endogenous SA contents. However, exogenous α-ionone had no effect on the accumulation of SA (Supplementary Figure S2).

**Figure 4:**
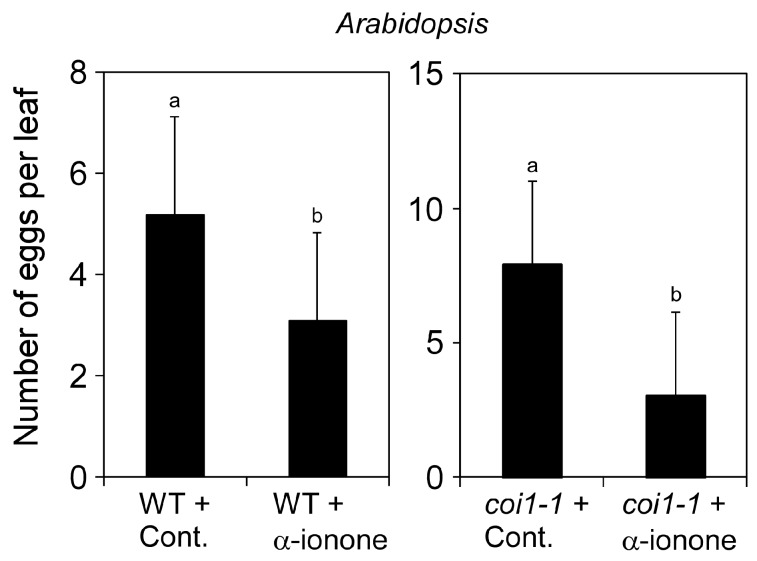
Analysis of the involvement of jasmonic acid in α-ionone-induced herbivore resistance using *Arabidopsis*. *Arabidopsis* wild-type (Col-0) or *coi1-1* leaves treated with 300 μM α-ionone or solvent alone (Cont.) for 48 h were used for the infestation assay using western flower thrips. Female thrips were placed on the leaf surface for 5 d, and the numbers of laid eggs were counted. Values are the mean ± SD (*n* = 10–12 replicates) Different letters indicate significant differences among treatments (*p* < 0.005, *t*-test).

To further examine whether JA is involved in α-ionone-induced resistance to western flower thrips, we used an *Arabidopsis* assay system. We confirmed whether α-ionone is effective for the combination of *Arabidopsis* and western flower thrips by releasing adult female thrips onto the leaf surface of wild-type (Col-0) plants that were treated with 300 μM α-ionone. α-Ionone decreased the number of eggs laid by female thrips on wild-type leaves (Figure 4; *p* < 0.005, t = 2.56, df = 18). coronatine-insensitive1-1 (*coi1-1*) is an *Arabidopsis* mutant that is defective in JA perception and has been shown to exhibit enhanced susceptibility to egg deposition by western flower thrips [26]. If α-ionone-induced resistance to western flower thrips is mediated by JA, the inhibition of egg deposition will not occur in *coi1-1* plants after a treatment with α-ionone. The pretreatment of *coi1-1* plants with α-ionone markedly decreased the number of eggs laid by the pest (Figure 4; *p* < 0.001, t = 3.85, df = 22).

### 2.3. Effects of α-Ionone on Other Herbivore Pests

**Figure 5:**
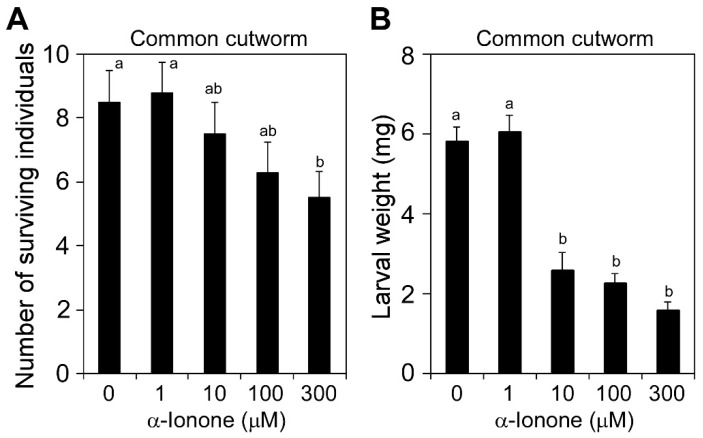
Effects of α-ionone on the infestation of tomato leaves by the common cutworm. Tomato leaves treated with different concentrations of α-ionone for 48 h were used for the infestation assay using the common cutworm. Hatchlings were released on the leaf surface, and the numbers (**A**) and weights (**B**) of surviving individuals were measured 5 d after the inoculation. Values are the mean ± SD (*n* = 10 replicates). Different letters indicate significant differences among treatments (*p* < 0.05, Tukey–Kramer HSD test).

We also examined the protective effects of α-ionone on other herbivore pests using several combinations of pests and their host plants, such as common cutworm–tomato, vegetable leafminer (*Liriomyza sativae*)–tomato, and brown planthopper (*Nilaparvata lugens*)–rice (*Oryza sativa*). In the combination of the common cutworm and tomato, hatchlings were released onto the surface of leaves that were treated by immersing into a solution containing different concentrations of α-ionone for 48 h. Resistance was assessed by measuring the numbers and weights of surviving larvae 5 d after the inoculation. The numbers (Figure 5A; *p* < 0.05, *F* = 4.7378) and weights (Figure 5B; *p* < 0.001, *F* = 40.510) of surviving larvae were decreased by α-ionone at 300 μM and 10–300 μM, respectively. In the other combination of the vegetable leafminer and tomato, we released pairs of adult males and females onto tomato plants that were treated with α-ionone vapor and assessed by counting the numbers of pupated individuals. No significant differences were observed in the number of pupated individuals between treatments (Supplemental Figure S3; *p* > 0.05, *F* = 0.962). In the combination of the brown planthopper and rice, we released nymphs on rice plants that were treated with α-ionone vapor in a sealed pot and assessed by counting the number of eclosed adults. No significant differences were observed in the number of eclosed individuals (Supplemental Figure S4A; chi-squared test, *p* = 0.84) or the day required for the completion of adult eclosion between treatments (Supplemental Figure S4B; chi-squared test, *p* = 0.87).

## 3. Discussion

When applied to tomato leaves, α-ionone decreased the survival rate of female western flower thrips and egg deposition by the same pest without exhibiting its insecticidal activity. This result suggests that reductions in the survival of female western flower thrips and egg deposition were due to defense responses induced in the plant after the treatment with α-ionone. Since *Arabidopsis coi1-1* plants, similar to wild-type plants, exhibited reduced egg deposition in response to α-ionone, COI-mediated JA signaling did not appear to be involved in α-ionone-induced resistance to western flower thrips. Loliolide was recently found to induce JA-independent resistance to multiple herbivore pests, including western flower thrips and the common cutworm [5]. However, exogenously applied α-ionone did not enhance the expression of *SlLin5*, a loliolide-responsive tomato gene. Furthermore, α-ionone exerted a positive effect on the induction of *SlGluB* expression, whereas loliolide did not [5]. These findings suggest that α-ionone and loliolide have different modes of action, at least, for inducing the expression of some defense-related genes.

Common cutworm larvae fed on α-ionone-treated tomato leaves exhibited a reduction in weight, suggesting that α-ionone affects the larval development of the common cutworm. This delay in larval development may be regarded as a consequence of the activation of the defense responses of plants against herbivores. For example, JA is known to affect the larval development of the common cutworm through the production of defense-related proteins, such as SlPin2 and SlLapA1 [27,28]. *SlPin2* and *SlLapA1* do not appear to be involved in the delay in the larval development of the common cutworm induced by α-ionone because α-ionone did not induce the expression of these two genes.

α-ionone also induced the expression of *SlChi9*, a tomato gene encoding basic chitinase. A previous study showed that the expression of *SlChi9* and *SlGluB* was induced in response to attacks by the tobacco whitefly or greenhouse whitefly [23]. Although chitinase and β-1,3-glucanase are well-known defense-related proteins for infection by pathogens, the role of these proteins in herbivore resistance remains unclear. Further studies to examine whether *SlChi9* and *SlGluB* are involved in α-ionone-induced herbivore resistance are required.

Although we found that exogenously applied α-ionone exerted a positive effect on the inhibition of infestation by western flower thrips and the common cutworm, this result is insufficient to demonstrate the actual role of α-ionone in herbivore resistance in plants. Molecular genetic studies using mutants or transgenic plants with an alteration in the endogenous α-ionone content are needed. However, no such mutant or plant has been reported to date. For example, a headspace analysis of volatile compounds released from *Arabidopsis* overexpressing *AtCCD1* revealed that a high level of β-ionone was detected in the headspace, whereas that of α-ionone was not [20]. This implies that α-ionone and β-ionone have different localizations and/or a gene other than *CCD1* involved in the synthesis of α-ionone exists in *Arabidopsis*. Further studies to clarify these issues are required.

Our results indicated that α-ionone was effective for western flower thrips in laboratory experiments. Since α-ionone is a volatile compound, it may evaporate and diffuse in an open field under certain weather conditions such as strong winds and high temperature. These chemical properties of α-ionone should be considered in field experiments.

## 4. Materials and Methods 

### 4.1. Plant Materials and Herbivores

Tomato (*S. lycopersicum* cv. Micro-Tom) plants were grown under 16 h light/8 h dark at 25 °C. *Arabidopsis* (*A. thaliana*) plants were grown under 10 h light/14 h dark at 22 °C. All *Arabidopsis* plants including *coi1-1* were in the Columbia (Col-0) background. *coi1-1* has been described previously [29]. Japonica rice (*O. sativa* cv. Koshihikari) plants were grown under 16 h light/8 h dark at 25 °C.

The western flower thrips, *F. occidentalis* (Pergande; Thysanoptera: Thripidae) has been described previously [5]. The eggs of *S. litura* (Fabricius; Lepidoptera: Noctuidae) and the vegetable leafminer (*L. sativae*) were purchased from a private company (Sumika Technoservice Co., Takarazuka, Japan). Brown planthoppers (*N. lugens*) were collected in Japan in 1966 and have been maintained in our organization (National Agriculture and Food Research Organization) under 16 h light/8 h dark at 25 °C.

### 4.2. Chemical Treatments

α-Ionone (Wako, Osaka, Japan), β-ionone (Wako), and β-cyclocitral (Alfa Aesar, Lancashire, UK) were dissolved in ethanol or methanol and diluted to appropriate concentrations. 

In the experiment shown in Figure 1, leaf discs (1 cm in diameter) were punched out from intact tomato (three to five leaf discs from one plant) and floated on a solution containing 0.8 mL of each compound diluted with water or 0.1% (*v*/*v*) methanol in one well of a 48-well polystyrene plate for 48 h. After removing the chemical solution with a pipette, 0.8 mL of distilled water was added to each well, and each plate was used for herbivore infestation assays.

In the vapor treatment of tomato plants, 4-week-old plants were placed in 1000-mL plastic pots together with microtubes containing 100 μL of different concentrations of α-ionone diluted with ethanol or ethanol alone as a control. Each pot was sealed with a plastic lid with one hole (7.5 cm × 6 cm) covered with a fine nylon mesh, covering the top of the cup with a plastic wrap, and incubated 25 °C for 24 h. After removing the microtubes, each pot was used for herbivore infestation assays, total RNA extraction, or phytohormone measurements. Fifteen rice seeds were grown in 500-mL plastic pots for 7 days, exposed to α-ionone for 72 h in a similar manner, and then used for brown planthopper infestation assays.

In the experiment shown in Figure 5, tomato leaves were excised with scissors from the 3–5 leaf positions of plants and floated on α-ionone diluted with water or 0.1% methanol alone as a control in a glass dish at 25 °C for 48 h. After briefly washing with distilled water to remove the chemical solution, each leaf was used for the assay with the common cutworm. 

### 4.3. Herbivore Infestation Assays

In the experiments shown in Figure 1 and Figure 4, one female western flower thrips per leaf disc was released on the leaf surface. The plate was covered with a plastic film (ABI Prism Optical Adhesive Cover, Applied Biosystems, USA), and seven small holes per well were punctuated with a 27G injection needle for ventilation. The plate was incubated at 25 °C for 3 d for tomato and 5 d for *Arabidopsis*. Leaf discs were stained with trypan blue as described previously [30], and the numbers of stained eggs were counted. We regarded the combination of one female and one leaf disc as one biological replicate.

In the assay using western flower thrips and α-ionone vapor-treated tomato, 20 adult females per plant were released on one tomato plant, and each pot was sealed with a plastic lid without covering the top of the cup with a plastic wrap and incubated at 25 °C. The numbers of surviving individuals were counted 14 d after the inoculation. We regarded the combination of 20 females and one plant as one biological replicate.

In the assay using the common cutworm, 10 hatchlings were released onto the surface of one tomato leaf with a petiole that was inserted into a 1.5-mL microtube filled with distilled water to prevent water loss from the leaf during the incubation and then incubated in a sealed plastic cup (9 cm in diameter and 14 cm in height) at 25 °C. The numbers and weight of surviving individuals was measured 5 d after the inoculation. We regarded the combination of 10 hatchlings and one tomato leaf as one biological replicate and used 10 replicates for each chemical.

In the assay using the vegetable leafminer, one pair of an adult male and female was released on one tomato plant in a 1000-mL plastic pot. Each pot was sealed with a plastic lid without covering the top of the cup with a plastic wrap and incubated at 25 °C. The numbers of pupated individuals were counted 14 d after the inoculation. We regarded the combination of one mating pair and one plant as one biological replicate.

In the assay using the brown planthopper, 30 second-instar nymphs per 15 rice plants were released near the plant in a plastic pot. Each pot was covered with a plastic lid and incubated at 25 °C. Individuals successfully located on rice plants the day after the release were used for subsequent observations. Observations were performed by recording the survival and developmental stages of individuals every day until all individuals eclosed or died. The assay was repeated three times.

### 4.4. Insecticidal Activity Assays

Second-instar larvae of western flower thrips were dropped into a solution containing 300 μM α-ionone diluted with water or 0.1% (*v*/*v*) methanol alone as a control for 5 sec, and one larva per leaf was placed on the surface of tomato leaves confined within modified Munger cells [5] and incubated at 23 °C under 16 h light/8 h dark. The numbers of surviving individuals were counted 48 h after the inoculation. We used 30 larvae for each chemical.

### 4.5. Total RNA Extraction and Quantitative Real-Time PCR

Nine leaf discs (8 mm in diameter) were harvested from three tomato plants and used for the extraction of total RNA. We regarded nine leaf discs as one biological replicate and used three replicates for each chemical concentration. Extraction and purification were performed using the RNeasy Plus Mini Kit (Qiagen) in accordance with the manufacturer’s instructions.

A quantitative real-time PCR analysis using total RNA was performed in a two-step reaction using a SYBR Green kit (Bio-Rad) as described previously [25]. Information on the primers used is shown in Supplemental Table S1. The expression levels of *Slactin* were used to normalize those of the target genes. 

### 4.6. Phytohormone Measurements

Six leaf discs (8 mm in diameter) were punched out from three tomato plants and used for the extraction of JA and SA. We regarded six leaf discs as one biological replicate and used three replicates for each chemical concentration. The extraction and quantification of JA and SA were performed as described previously [31].

### 4.7. Statistical Analyses

We used Fisher’s exact probability test to compare survival rates in the dipping assay of Figure 2B. These analyses were conducted using R version 3.3.3 [32]. Differences in the number of trypan blue-stained eggs or emerging individuals (Figure 1), the number of surviving individuals (Figure 2A and Figure 5A), and larval weights (Figure 5B) were tested by a one-way analysis of variance (ANOVA) and then compared using the Tukey–Kramer honestly significant difference (HSD) test using JMP version 9.0.2 (SAS Institute Inc. Cary, NC, USA). The Student’s *t*-test was used to compare the significance of the difference in the mean of two samples.

## Supplementary Materials

The following are available online, Figure S1: Experimental set-up for the treatment of tomato plants with α-ionone vapor, Figure S2: Effects of α-ionone on the accumulation of jasmonic acid and salicylic acid in tomato leaves, Figure S3: Effects of α-ionone on the infestation of tomato leaves by the vegetable leafminer, Figure S4: Effects of α-ionone on the infestation of rice leaves by the brown planthopper, Table S1: List of primers used in the present study.
