# Supplementary material for: α-Ionone, an Apocarotenoid, Induces Plant Resistance to Western Flower Thrips, Frankliniella occidentalis, Independently of Jasmonic Acid
Source: Molecules. 2019 Dec 19;25(1):17. doi: 10.3390/molecules25010017 (PMC6982998; doi:10.3390/molecules25010017)
Supplement: Supplementary file 1 [file molecules-25-00017-s001.zip › molecules-665017-supplementary.pptx]

## Slide 1
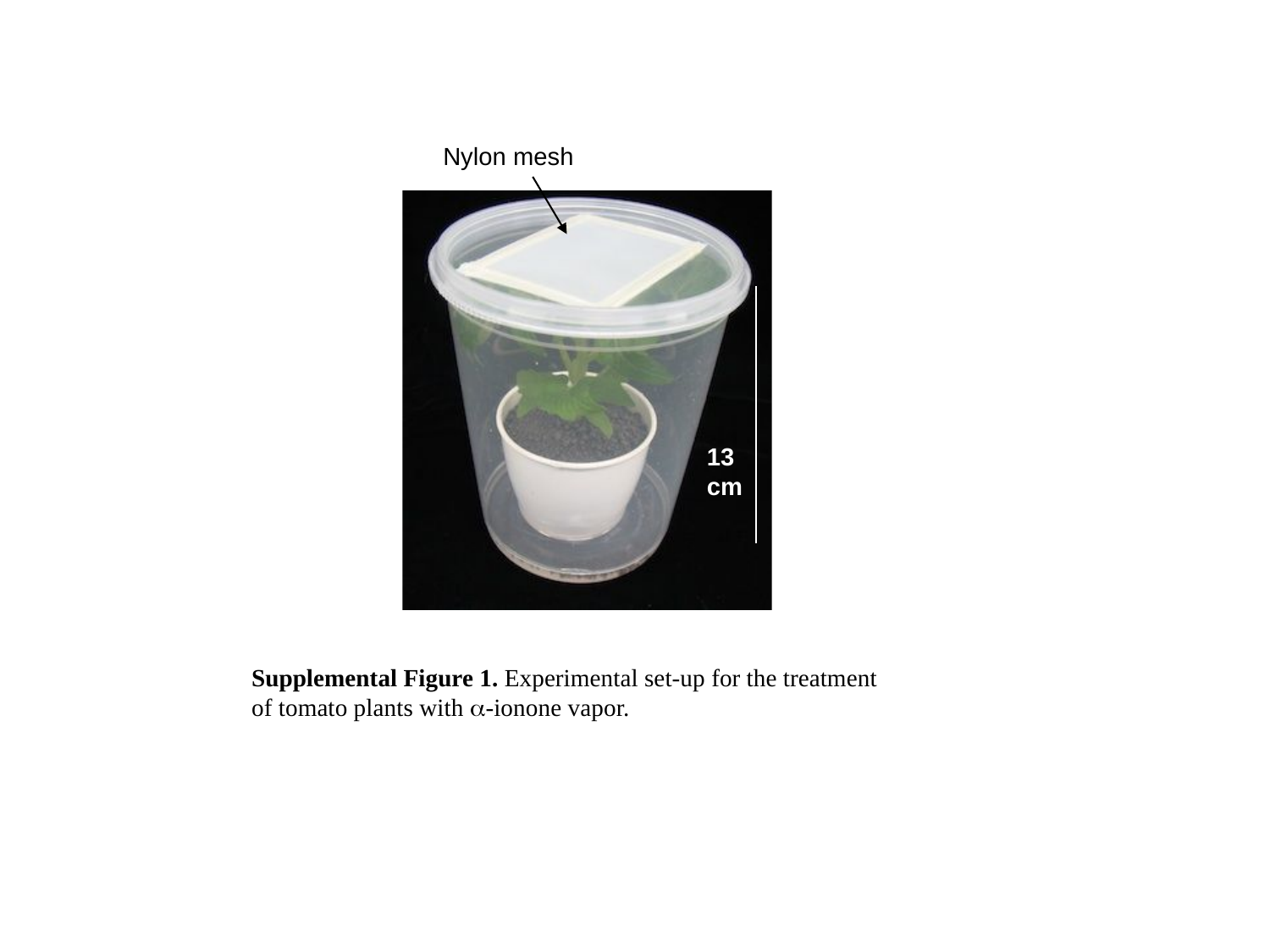

Nylon mesh
13
cm
Supplemental Figure 1. Experimental set-up for the treatment
of tomato plants with a-ionone vapor.

## Slide 2
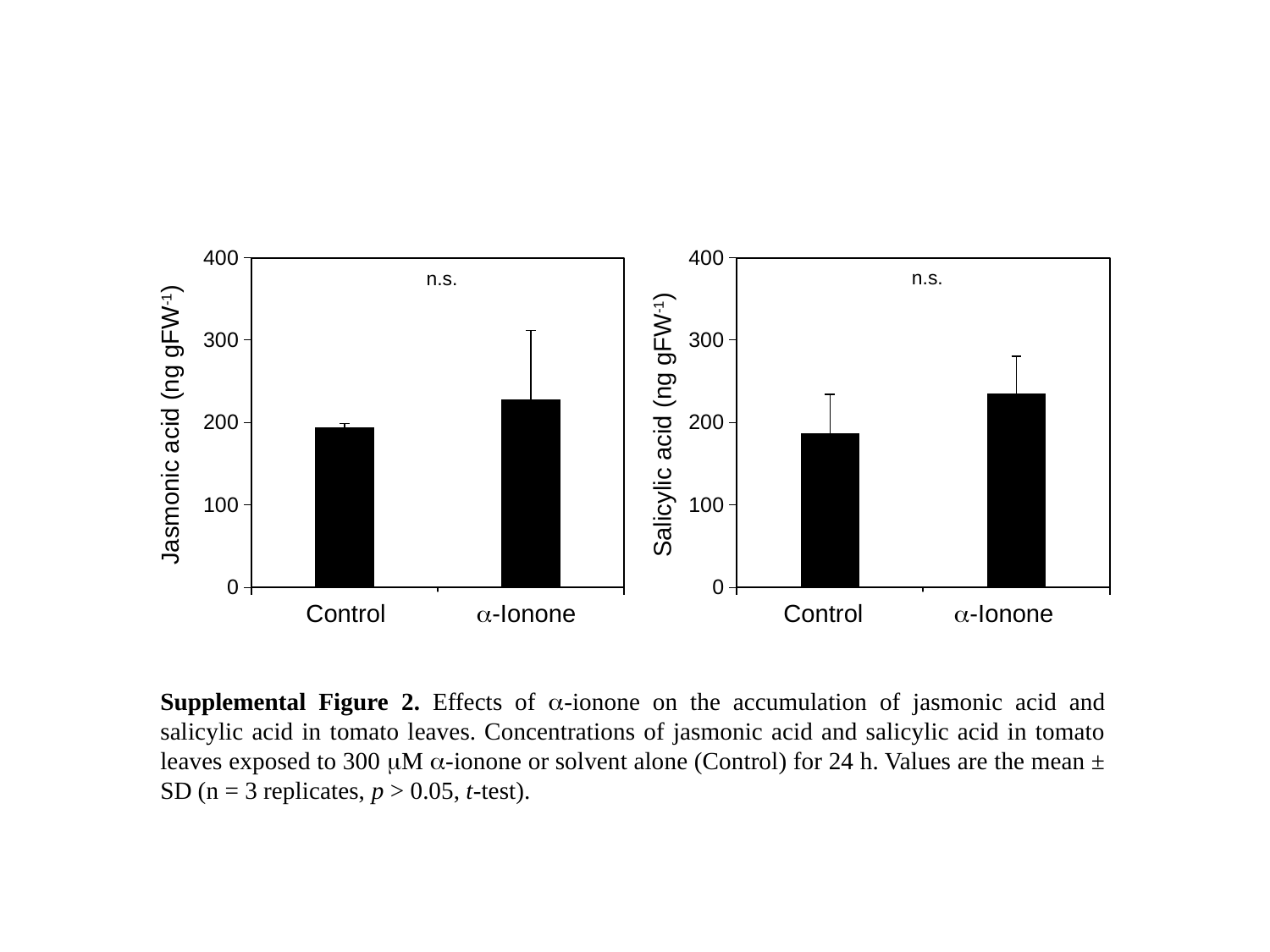

### Chart
| Category | |
|---|---|
### Chart
| Category | |
|---|---|n.s.
n.s.
Jasmonic acid (ng gFW-1)
Salicylic acid (ng gFW-1)
Control a-Ionone
Control a-Ionone
Supplemental Figure 2. Effects of α-ionone on the accumulation of jasmonic acid and salicylic acid in tomato leaves. Concentrations of jasmonic acid and salicylic acid in tomato leaves exposed to 300 μM α-ionone or solvent alone (Control) for 24 h. Values are the mean ± SD (n = 3 replicates, p > 0.05, t-test).

## Slide 3
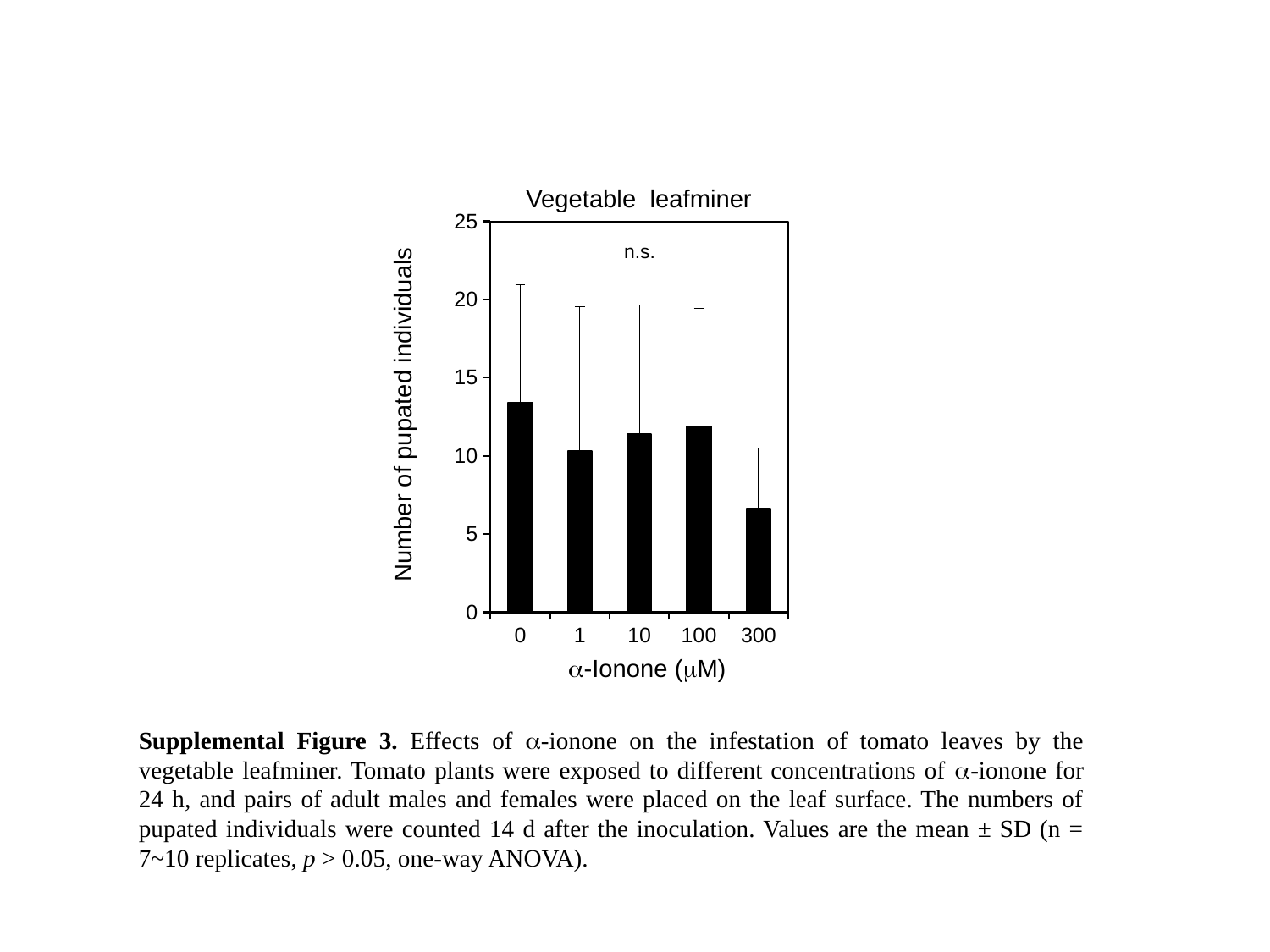

Vegetable leafminer
### Chart
| Category | |
|---|---|
| 0 | 13.38 |
| 1 | 10.29 |
| 10 | 11.4 |
| 100 | 11.89 |
| 300 | 6.63 |n.s.
Number of pupated individuals
a-Ionone (mM)
Supplemental Figure 3. Effects of a-ionone on the infestation of tomato leaves by the vegetable leafminer. Tomato plants were exposed to different concentrations of a-ionone for 24 h, and pairs of adult males and females were placed on the leaf surface. The numbers of pupated individuals were counted 14 d after the inoculation. Values are the mean ± SD (n = 7~10 replicates, p > 0.05, one-way ANOVA).

## Slide 4
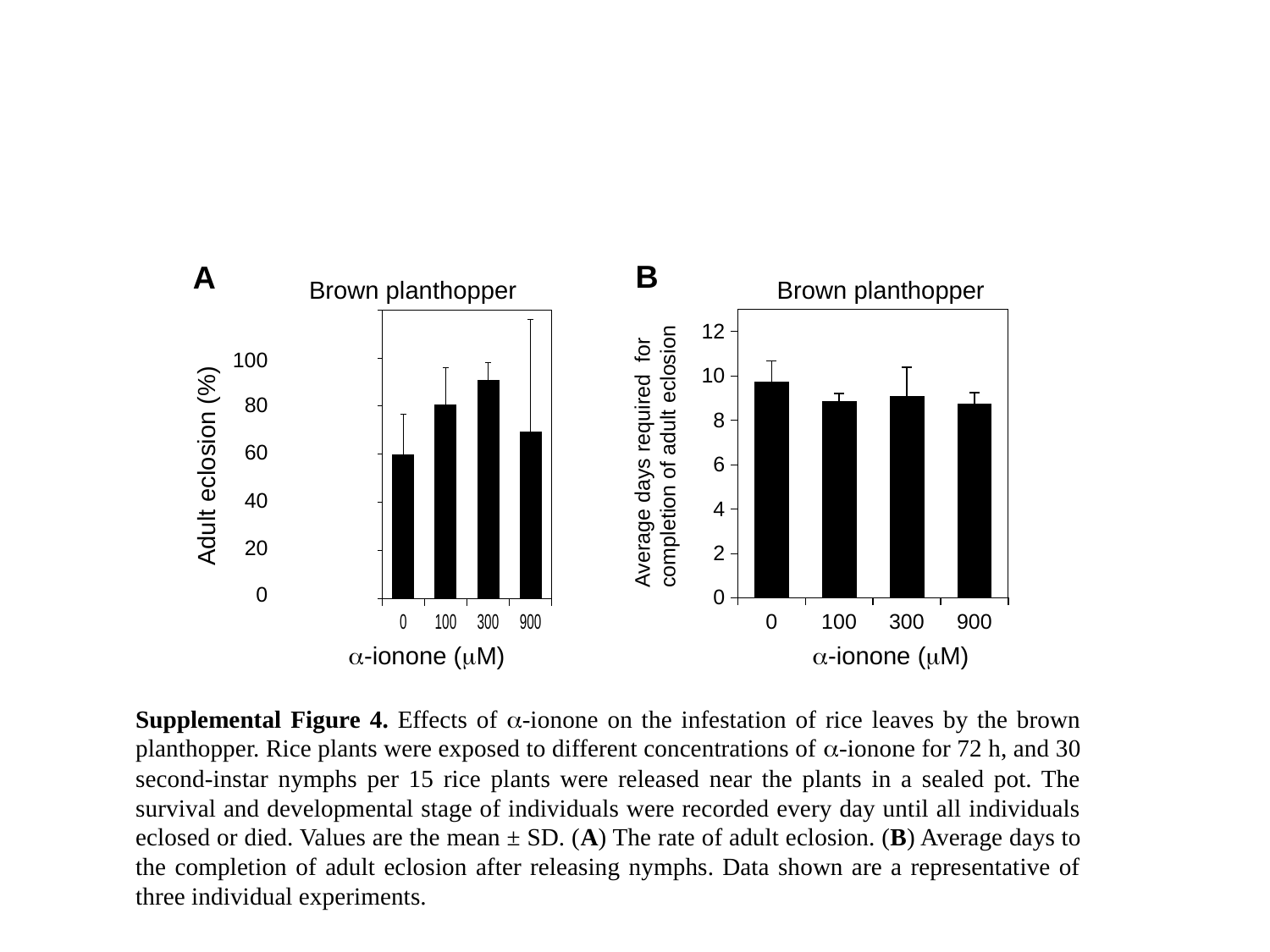

B
A
Brown planthopper
Brown planthopper
### Chart
| Category | |
|---|---|
| 0 | 0.5984126984126984 |
| 100 | 0.8041871921182265 |
| 300 | 0.9051724137931035 |
| 900 | 0.6932234432234433 |
### Chart
| Category | |
|---|---|
| 0 | 9.722222222222221 |
| 100 | 8.842105263157896 |
| 300 | 9.076923076923077 |
| 900 | 8.75 |100
80
Average days required for
completion of adult eclosion
60
 Adult eclosion (%)
40
20
0
a-ionone (mM)
a-ionone (mM)
Supplemental Figure 4. Effects of a-ionone on the infestation of rice leaves by the brown planthopper. Rice plants were exposed to different concentrations of a-ionone for 72 h, and 30 second-instar nymphs per 15 rice plants were released near the plants in a sealed pot. The survival and developmental stage of individuals were recorded every day until all individuals eclosed or died. Values are the mean ± SD. (A) The rate of adult eclosion. (B) Average days to the completion of adult eclosion after releasing nymphs. Data shown are a representative of three individual experiments.

## Slide 5
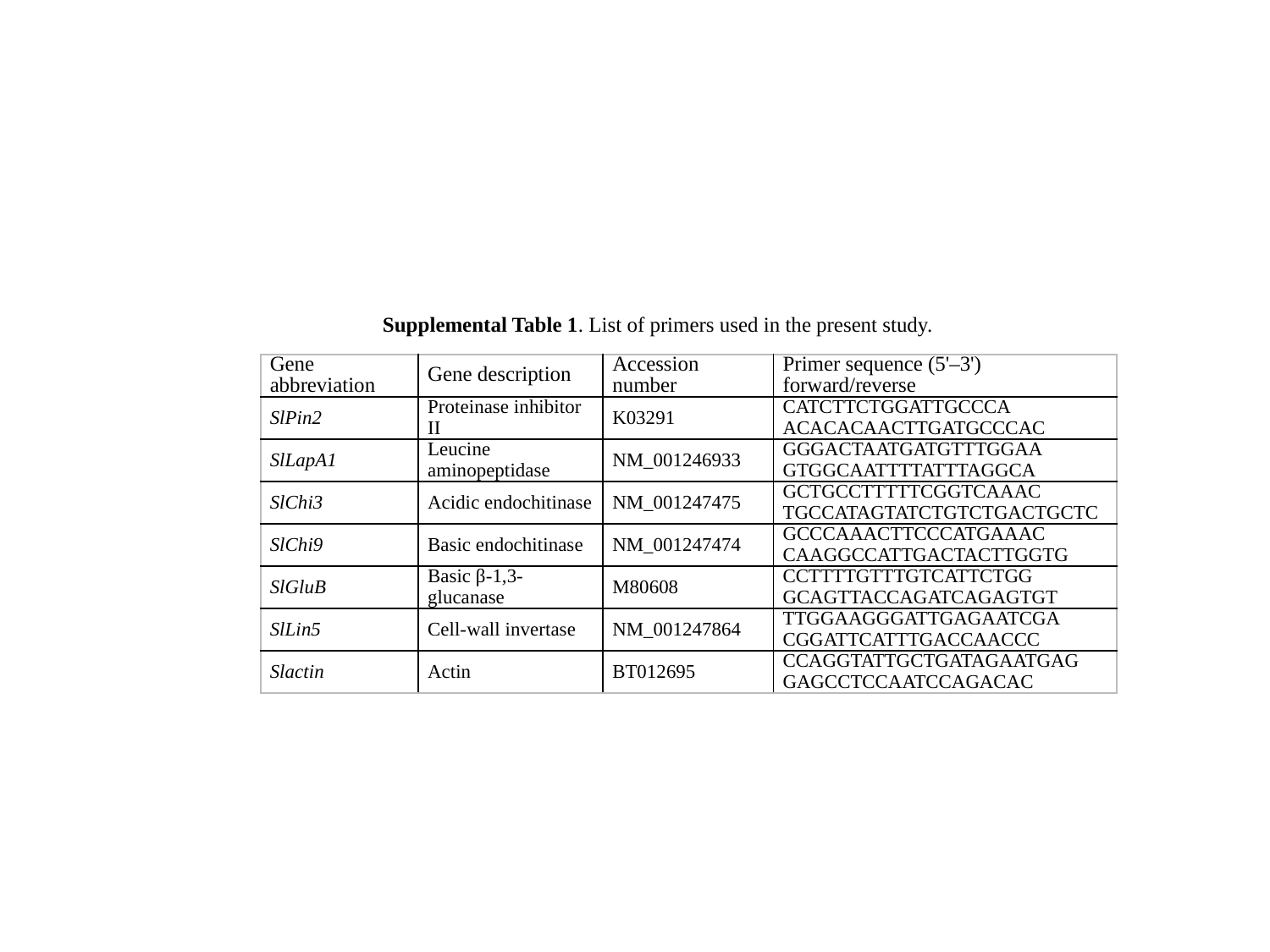

Supplemental Table 1. List of primers used in the present study.
| Gene abbreviation | Gene description | Accession number | Primer sequence (5'–3') forward/reverse |
| --- | --- | --- | --- |
| SlPin2 | Proteinase inhibitor II | K03291 | CATCTTCTGGATTGCCCA ACACACAACTTGATGCCCAC |
| SlLapA1 | Leucine aminopeptidase | NM\_001246933 | GGGACTAATGATGTTTGGAA GTGGCAATTTTATTTAGGCA |
| SlChi3 | Acidic endochitinase | NM\_001247475 | GCTGCCTTTTTCGGTCAAAC TGCCATAGTATCTGTCTGACTGCTC |
| SlChi9 | Basic endochitinase | NM\_001247474 | GCCCAAACTTCCCATGAAAC CAAGGCCATTGACTACTTGGTG |
| SlGluB | Basic β-1,3-glucanase | M80608 | CCTTTTGTTTGTCATTCTGG GCAGTTACCAGATCAGAGTGT |
| SlLin5 | Cell-wall invertase | NM\_001247864 | TTGGAAGGGATTGAGAATCGA CGGATTCATTTGACCAACCC |
| Slactin | Actin | BT012695 | CCAGGTATTGCTGATAGAATGAG GAGCCTCCAATCCAGACAC |
